# Multiple Giant Stones in an Augmented Bladder: A Case Report

**DOI:** 10.7759/cureus.81430

**Published:** 2025-03-29

**Authors:** Mehmet K Asutay, Celal Kilinc, Osman Bozkurt, Kutsan Coktu, Serdar Celik

**Affiliations:** 1 Urology, Mersin Tarsus State Hospital, Mersin, TUR

**Keywords:** bladder dysfunction, giant bladder stone, struvite stones, urinary bladder stone, urinary stone disease

## Abstract

Bladder stones are a common finding in urology practice although they are less common when compared to kidney and ureteral stones. This case report involves a male patient in his 40s with multiple giant bladder stones. The patient had neurogenic bladder disease due to spina bifida and had a history of bladder augmentation with continent urinary diversion when he was a child. He presented with difficulty in emptying bladder with clean intermittent catheterization. Open cystolithotomy was performed and six giant stones, five pyramid-shaped stones and a cube-shaped stone with a maximum diameter of 7 cm, were removed from the bladder. In our opinion, the rare occurrence of a high stone burden, stone weight and size, and geometric shapes of stones makes this case interesting. The patient was discharged from the hospital on the 10th day after the surgery and there were no postoperative complications.

## Introduction

Bladder stones are frequently seen in daily urology practice though they are less common than kidney and ureteral stones. Hematuria, discomfort and difficulty during urination are most common symptoms of bladder stones. Patients with neurogenic bladder are at an increased risk of bladder stones, potentially due to chronic bacteriuria and incomplete emptying of bladder [1]. Available data shows that the bladder augmentation procedure is highly effective at protecting the upper urinary tract in patients with neurogenic bladder, but complications such as bladder stones, chronic infections, and malignancy, are also common [2]. Bladder stones can be treated either with endoscopic procedures or open cystolithotomy. Here, we report a case of a patient with neglected neurogenic bladder disease who presented with six giant geometrically shaped bladder stones. Open cystolithotomy was the preferred treatment method. To our knowledge, stone burden and stone size reported here are the highest values recorded so far in the literature.

## Case presentation

A patient in his 40s with spina bifida presented to our clinic with difficulty during clean intermittent catheterization. He had a history of bladder augmentation and appendicovesical continent diversion procedure done when he was a child. He had no motor deficit. After the augmentation surgery, he did not come for routine urologic checkups. There were no symptoms of pain, hematuria, or fever. A mild tenderness of the lower abdomen was seen on physical examination, but guarding or rebound symptoms were not seen.

**Figure 1:**
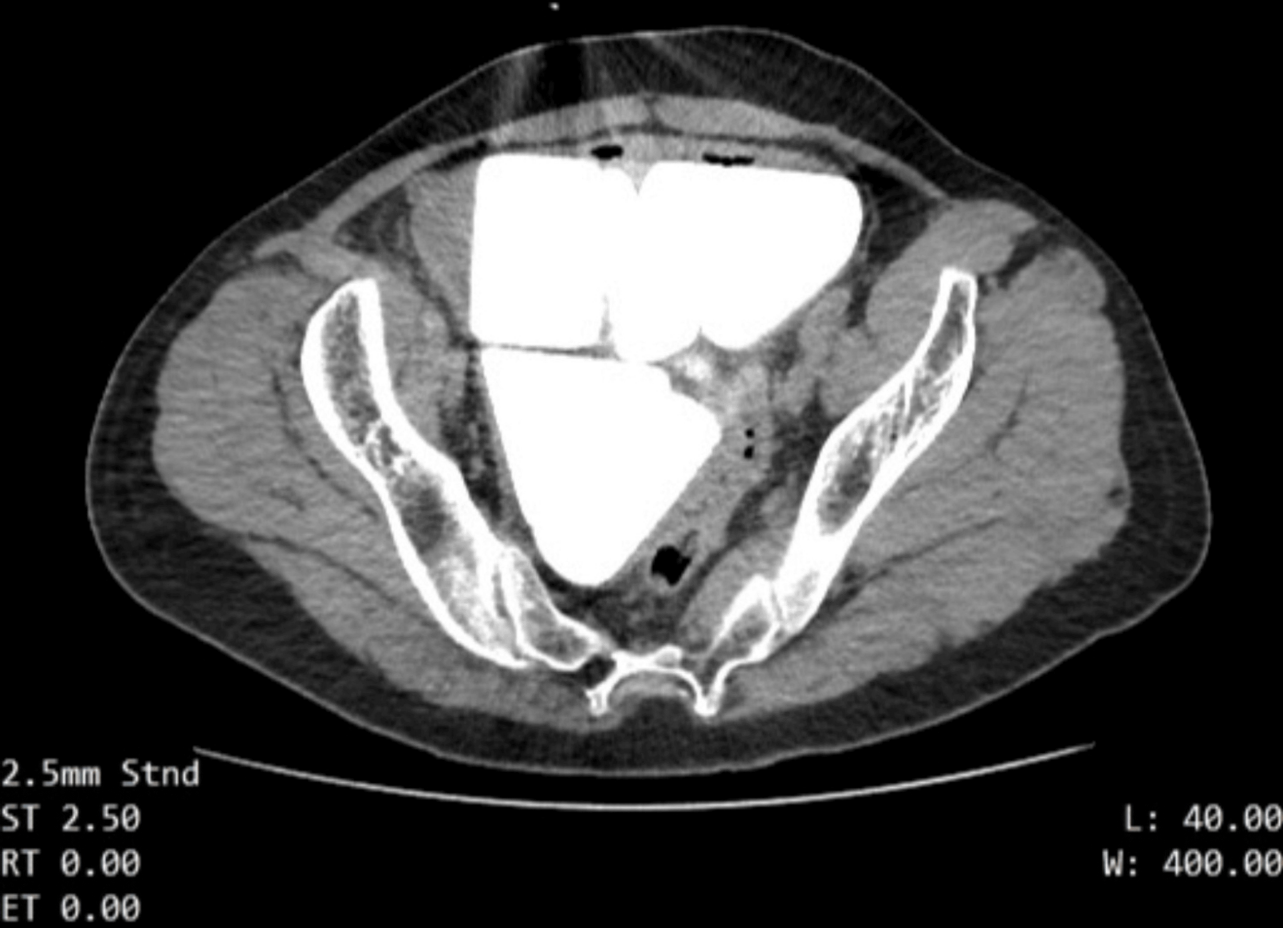
An axial CT image of bladder stones

**Figure 2:**
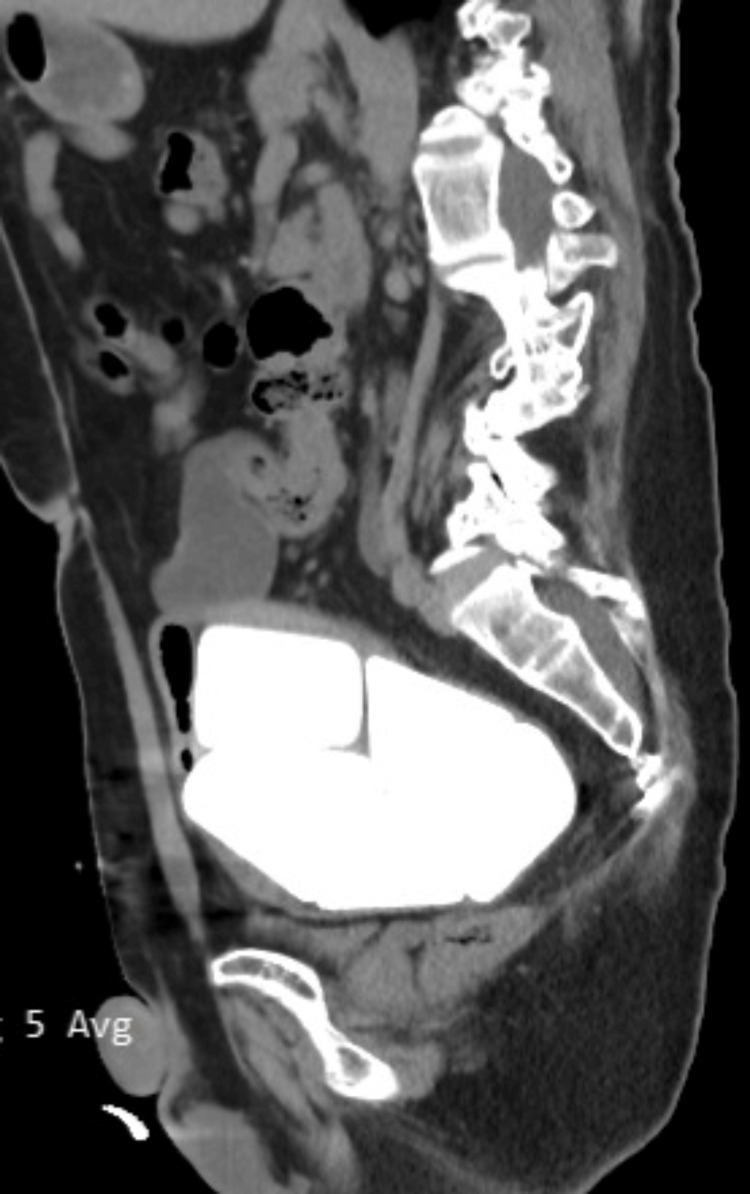
A sagittal CT image of bladder stones

A urinary ultrasound was performed, and giant bladder stones were reported. A CT scan of the abdomen showed six giant bladder stones with geometrical shapes (Figures 1, 2). The serum creatinine level was 0.9 mg/dl; the urine culture was positive for extended-spectrum beta-lactamase (ESBL)-producing Klebsiella. The patient was hospitalized and had ertapenem 1000 mg intravenously per day for seven days. The control urine culture was sterile. Due to the massive stone burden, endoscopic laser treatment was not preferred and an open cystolithotomy procedure was planned.

**Figure 3:**
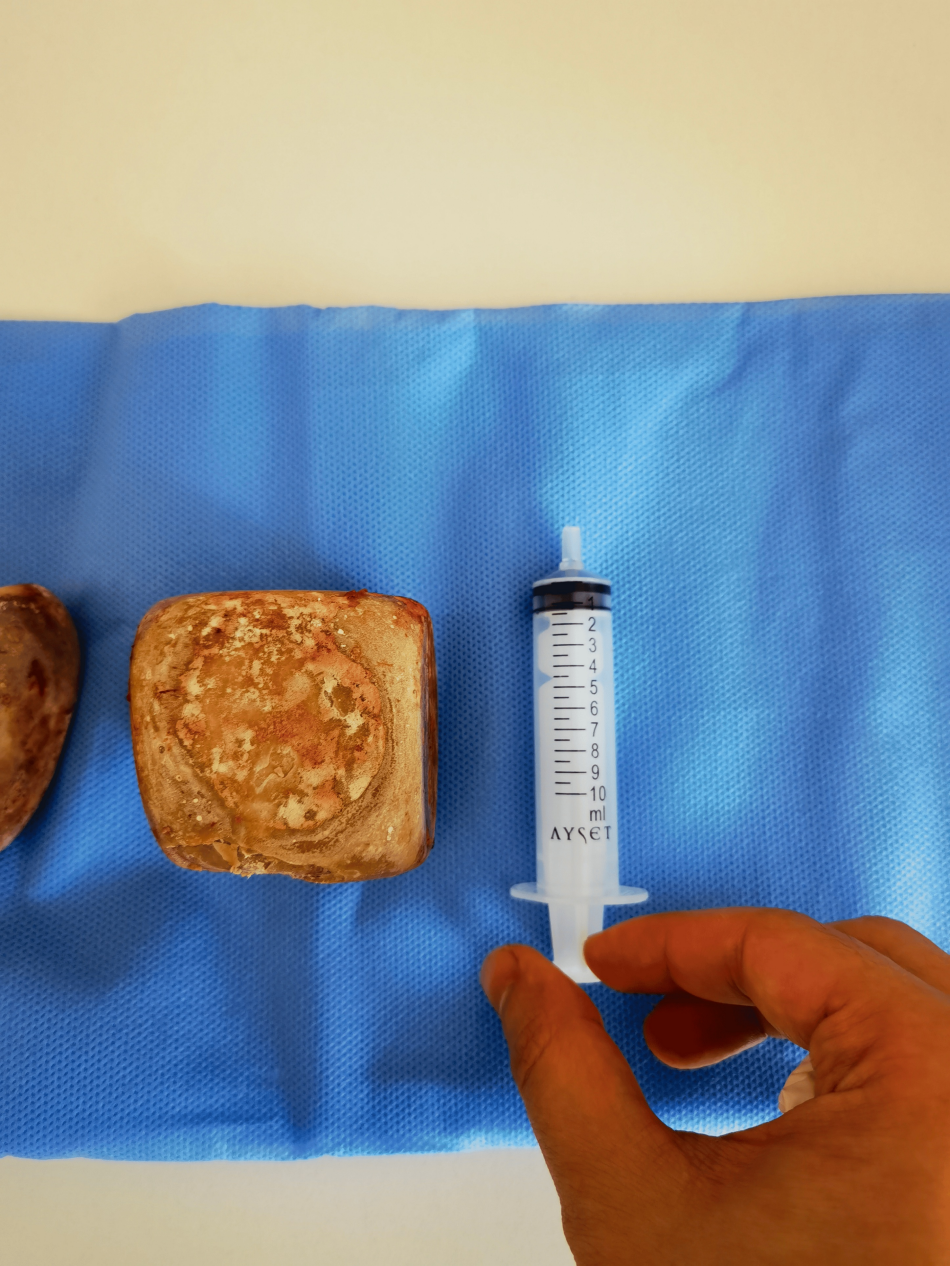
Cube-shaped bladder stone measuring 7x7x7 cm

**Figure 4:**
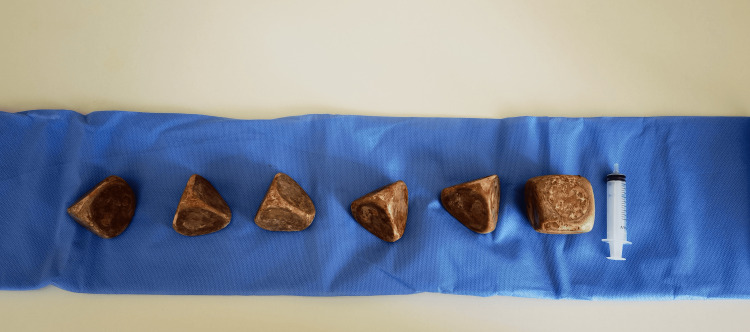
Five pyramid-shaped stones and one cube-shaped stone

Cystolithotomy was performed as planned. Five pyramid-shaped stones and one cube-shaped stone were taken out from the bladder. The cube-shaped stone measured 7x7x7 cm, whereas the pyramid-shaped ones measured 6x6x6.5 cm, 5.8x6x6.2 cm, 6.2x6x5.8 cm, 5.7x5.9x6 cm, and 6.2x6.7x5.8 cm, respectively (Figures 3, 4). The stones weighed 2100 grams in aggregate.

A foley catheter was placed via continent diversion and the patient also had a Hemovac drain catheter for five days. On the 10th day, the foley catheter was removed and the patient was discharged without any complications. At the follow-up, there were no complications and the patient had no difficulty doing clean intermittent catheterization. He was told to perform bladder irrigation two or three times a week and come to the urology outpatient clinic for routine urologic controls. Neither prior (or pre-surgical) nor follow-up (or post-surgical) urine cultures grew urease-producing bacteria. The X-ray analysis of the stone revealed a magnesium ammonium phosphate (struvite) stone. No new bladder stones were observed during routine follow-ups, but solid mucus particles were sometimes seen during irrigation.

## Discussion

Bladder stones constitute 5% of urinary system stones and are 4 to 10 times more common in men [2]. Primary bladder stones can be seen frequently in children without an underlying condition in the presence of factors such as malnutrition and chronic diarrhea [3]. Secondary bladder stones occur in the presence of an underlying urological abnormality such as bladder outlet obstruction, neurogenic bladder or history of bladder augmentation [4]. Calcium oxalate and calcium phosphate stones are the most common secondary bladder stones followed by magnesium ammonium phosphate stones [4].

There are several treatment methods for bladder stones such as chemolysis, shockwave lithotripsy, endoscopic/percutaneous cystolithotripsy, and laparoscopic/robotic/open cystolithotomy [5]. Although open cystolithotomy is an effective method, the duration of hospital stay and catheterization is relatively longer when compared to other methods [5]. The patient mentioned in this case report had many underlying factors such as neurogenic bladder, chronic bacteriuria, bladder augmentation and entero-cysto-vesical diversion (Mitrofanoff). The risk of stone formation in patients with neurogenic augmented bladder with continent diversion is quite high, with stone formation rates reaching up to 40% [6]. Our patient had no bladder sensation, and he neglected his regular controls, which explains the giant size of stones. Excessive mucus production, inadequate emptying of bladder during clean intermittent catheterization, presence of urease-producing bacteria in the urine and continent diversions are factors related to bladder stone formation; regular irrigation of bladder may prevent the formation of stones [7,8]. There are various studies in the literature that mention large bladder stones. What makes this case report different is the patient's stone burden, the total weight and size of the stones, and the interesting geometric shapes [9,10]. Due to the lack of regular urological follow-ups, the existing stones grew in the augmented bladder for many years reaching gigantic dimensions, which is very rare and may be a record in the literature in terms of the total size and weight.

## Conclusions

Although the treatment choice for bladder stones is debatable, open cystolithotomy appears to be a valid option for the treatment of giant bladder stones in the endourological era. Augmented neurogenic bladder patients are more prone to have bladder calculi. Patients with augmented neurogenic bladder should be followed up routinely, and the upper and lower urinary systems should be checked regularly, failing which bladder stones can reach gigantic sizes just like seen in this case.

## References

[1] Zhang W, Shen R, Shang Z (2024). Incidence of and risk factors for urinary stones among patients with spinal cord injury: a systematic review with meta-analysis. Eur Urol Open Sci.

[2] Hoen L, Ecclestone H, Blok BF (2017). Long-term effectiveness and complication rates of bladder augmentation in patients with neurogenic bladder dysfunction: a systematic review. Neurourol Urodyn.

[3] Schwartz BF, Stoller ML (2000). The vesical calculus. Urol Clin North Am.

[4] Philippou P, Moraitis K, Masood J, Junaid I, Buchholz N (2012). The management of bladder lithiasis in the modern era of endourology. Urology.

[5] Takasaki E, Suzuki T, Honda M, Imai T, Maeda S, Hosoya Y (1995). Chemical compositions of 300 lower urinary tract calculi and associated disorders in the urinary tract. Urol Int.

[6] Donaldson JF, Ruhayel Y, Skolarikos A (2019). Treatment of bladder stones in adults and children: a systematic review and meta-analysis on behalf of the European Association of Urology Urolithiasis Guideline Panel. Eur Urol.

[7] Kelly JD, Keane PF (1998). Long-term results and complications of augmentation ileocystoplasty for idiopathic urge incontinence in women. Br J Urol.

[8] Hensle TW, Bingham J, Lam J, Shabsigh A (2004). Preventing reservoir calculi after augmentation cystoplasty and continent urinary diversion: the influence of an irrigation protocol. BJU Int.

[9] Mosa MM, Alsayed-Ahmad ZA, Razzouk Q, Alhmaidy O, Ismaeel H, Al Ahmad Y (2024). Extraction of a giant bladder stone (560 g) in a young female: a case report. Int J Surg Case Rep.

[10] Nugroho EA, Junita D, Wijaya YH (2019). Giant bladder stone with history of recurrence urinary tract infections: a rare case. Urol Case Rep.

